# Synergistic effects of the combined use of human‐cultured periosteal sheets and platelet‐rich fibrin on bone regeneration: An animal study

**DOI:** 10.1002/cre2.71

**Published:** 2017-08-02

**Authors:** Makoto Horimizu, Takehiko Kubota, Tomoyuki Kawase, Masaki Nagata, Mito Kobayashi, Kazuhiro Okuda, Koh Nakata, Hiromasa Yoshie

**Affiliations:** ^1^ Division of Periodontology, Department of Oral Biological Science Niigata University Graduate School of Medical and Dental Sciences Niigata Japan; ^2^ Division of Dental Pharmacology, Department of Oral Biological Science Niigata University Graduate School of Medical and Dental Sciences Japan; ^3^ Division of Oral and Maxillofacial Surgery, Department of Oral Biological Science Niigata University Graduate School of Medical and Dental Sciences Japan; ^4^ Bioscience Medical Research Center Niigata University Medical and Dental Hospital Japan

**Keywords:** biomaterial(s), bone regeneration, growth factors, tissue engineering

## Abstract

A human‐cultured alveolar bone‐derived periosteal (hCP) sheet is an osteogenic grafting material used clinically in periodontal regenerative therapy, while platelet‐rich fibrin (PRF), a platelet concentrate with fibrin clot, is considered to augment the wound healing process. Therefore, whether the combined use of hCP‐PRF complex could facilitate bone regeneration synergistically was evaluated in animal models. Human periosteal segments (1 × 1 mm) were cultured initially on plastic dishes and formed an hCP sheet. The hCP sheet was implanted with freshly prepared human PRF into subcutaneous tissue (hCP: n = 4, hCP + PRF: n = 4) and 4 mm diameter calvarial bone defect models (hCP: n = 4, hCP + PRF: n = 4, control [defect‐only]: n = 4) that prepared in nude mice. At 4 weeks postimplantation, new bone formation was evaluated by using μCT. Cell growth and neovascularization were evaluated by histochemical and immunohistological methods. In the subcutaneous tissue, mineral deposit formation, collagen deposition, and number of vessels were higher in the hCP + PRF group than in the hCP alone group. In the calvarial defect models, new bone formation was significantly higher in the hCP + PRF group than in the hCP alone group and defect‐only control group. The numbers of vessels and PCNA‐positive cells in calvarial defects were also increased in the hCP + PRF group more than in the hCP alone group. Platelet‐rich fibrin preparations support the proliferation and the growth of periosteal cells to form well‐combined active biological materials. Platelet‐rich fibrin also stimulates the local angiogenesis in the implantation site. Therefore, the combined use of hCP and PRF could be clinically applicable in bone regeneration therapy.

## 1 INTRODUCTION

Periodontal and alveolar bone regenerative therapies have been developed from bone grafting, guided tissue/bone regeneration, enamel matrix derivatives, and their use in combined therapies (Koop et al, 2012). In the last few decades, cell‐based grafting materials have been applied to bone regenerative therapies by using multipotent cells such as mesenchymal stem cells (MSCs) that can differentiate into a variety of cell types (Zomorodian and Baghaban Eslaminejad, 2012). Among reported cell sources of MSCs, such as bone marrow, periodontal ligament, dental pulp, and periosteum, we have been especially interested in periosteal cells (Kawase et al, 2010; Uematsu et al, 2013). Periosteum is easy to access intraorally by periodontists to prepare human‐cultured periosteal (hCP) sheets by periosteum explant cultures (Kawase et al, 2009). We have previously demonstrated that hCP sheets contain MSCs with osteogenic potency and produce various growth factors and cytokines involved in bone metabolism (Kawase et al, 2010; Uematsu et al, 2013). Clinically, it was also reported that autologous grafting of hCP sheets was one of the promising methods for treatment of alveolar bone atrophy and periodontal intrabone defects (Nagata et al, 2012; Okuda et al, 2009; Yamamiya et al, 2008). Therefore, hCP sheets are considered to be effective osteogenic grafting materials for periodontal regeneration therapy.

Enhancing the osteogenic activity of hCP sheets, combining hCP sheets and platelet‐rich plasma (PRP) improved clinical outcomes (Nagata et al, 2012; Okuda et al, 2009; Yamamiya et al, 2008). Platelet‐rich plasma is an autologous source of platelets and growth factors obtained from the peripheral blood of each patient, and PRP contains several different growth factors related to bone regeneration, eg platelet‐derived growth factor (PDGF) and transforming growth factor beta (TGF‐β), and it has been applied in plastic and maxillofacial surgery, as well as periodontal regeneration surgery (Kanthan et al, 2011; Pieri et al, 2009; Plachokova et al, 2008). PRP has been shown to enhance soft tissue regeneration, such as the formation of connective tissue and blood vessels (Nakajima et al, 2012; Okuda et al, 2003), but the liquid form of PRP has a disadvantage for grafting: PRP preparations need to be converted from their liquid form to a gel form by adding bovine thrombin before grafting (Kawase, 2015).

Alternatively, platelet‐rich fibrin (PRF) is a platelet concentrate in the form of fibrin clot and contains platelets, white blood cells, and growth factors (Dohan et al, 2006). The preparation of PRF is simpler than that of PRP and does not require anticoagulants. Furthermore, the fibrin gel maintains its form and release of growth factors at the application site. The amounts of growth factors in PRF are similar to those in PRP (Su et al, 2009); PDGF and TGF‐β contained in PRF are known to promote the healing of connective tissue and bone via stimulation of collagen production (Bolander, 1992; Pierce et al, 1991). Platelet‐rich fibrin was reported to also be a useful material for in vitro cultivation of periosteal cells as a scaffold (Gassling et al, 2010). However, the effects of PRF on osteogenic activity of grafting periosteal cells have still been unclear in cell‐based bone regeneration therapy.

Thus, we hypothesized that combined use of hCP sheets and PRF would be helpful for bone healing. With these applications, the combined use of PRF and hCP sheets could be a promising new method for bone regeneration, simultaneously promoting biological activity of bone regeneration. The aim of the present study was to elucidate the effect of the combined use of hCP sheets and PRF on bone regeneration as an engineered bone grafting material.

## 2 MATERIALS AND METHODS

### 2.1 Isolation and culture of human periosteal sheets

After obtaining informed consent, human periosteal tissue segments were aseptically dissected from the buccal side of the retromolar region where the alveolar bone was trimmed for extraction of impacted tooth in the mandibles of donors (Kawase et al, 2009). Small pieces of periosteum (1 × 1 mm) from 8 donors were placed on a 6‐well culture plate, and then explant culture was performed in humidified 5% CO_2_, 95% air at 37°C with medium 199 (Invitrogen, Carlsbad, CA, USA) supplemented with 10% fetal bovine serum (Invitrogen), 25 μg/mL l‐ascorbic acid, and antibiotics. Unless otherwise specified, hCP sheets were cultured in this growth medium for up to 28 days.

The study design and consent forms for all procedures performed with the study subjects were approved by the ethics committee for human subject use at Niigata University Medical and Dental Hospital in accordance with the Helsinki Declaration of 1975 and as revised in 2000.

### 2.2 Preparation of human platelet‐rich fibrin

Platelet‐rich fibrin was prepared as previously described (Kobayashi et al, 2012). In brief, blood was collected from 4 healthy volunteers, aged 28 to 33 (30.0 ± 2.7) years, using vacuum blood collection tubes, then centrifuged by a Medifuge centrifugation system (Silfradent Srl, Santa Sofia, Italy) using a program with the following characteristics: 3000 acceleration, 20 to 2700 rpm (600 g), 40 to 2400 rpm (500 g), 30 to 3000 rpm (800 g), and 3600 deceleration and stop.

The resulting PRF preparations were picked up, and the red thrombus (the fraction of red blood cells) was eliminated. The PRF was then compressed and trimmed into about a 1 mm thickness (8 × 8 mm) by using the PRF compression device and used for the following experiments.

### 2.3 Combining human‐cultured periosteal sheets with platelet‐rich fibrin

The hCP sheets at 14 days were rinsed 3 times by PBS, harvested from the dish by using a cell scraper preserving cell sheet form, and placed on trimmed PRF (1 × 8 × 8 mm) (hCP‐PRF complex) in the wet condition. Two milliliters of M199 medium were added to the dish, and the hCP‐PRF complex was incubated for 12 hours. After incubation, the hCP‐PRF complex was further cultured for 2 weeks. The medium was changed twice a week. The experimental setting is indicated at schemes in figure S1.

### 2.4 Animals and surgical procedure combining human‐cultured periosteal sheets with platelet‐rich fibrin

A total of 20 ICR nu/nu mice (male, 5 weeks old, weight: 20‐25 g) were divided into 2 groups as hCP (n = 4) and hCP + PRF (n = 4) for the subcutaneous implantation study and 3 groups as hCP (n = 4), hCP + PRF (n = 4), and defect‐only control (n = 4) for calvarial bone defect model. The mice were anesthetized with an intraperitoneal injection of pentobarbital. The surface of the surgical site was disinfected with povidone‐iodine solution before the operation. In the surgical procedure of implantation into the subcutaneous tissue, an incision was made in the mice's skin, and a dermal tissue layer was exfoliated like an envelope. Periosteal sheets at 28 days were rinsed 3 times with PBS and harvested with a cell scraper preserving cell sheet form. Periosteal sheets and fresh prepared PRF (8 × 8 mm) were implanted into the subcutaneous tissue of the nude mice as periosteal sheets positioned in the direction of the dermis, and the incisions were sutured.

Implantation into a calvarial bone defect was performed as follows. A subperiosteal incision was made on the scalp. A skin flap was retracted from the surgical site. Under irrigation with saline, a critical size bone defect was made on the midline using a trephine bur with a 4.0 mm outer diameter. A cylindrical polypropylene chamber was fitted on the bone defect and fixed to the calvarial bone by 2 micro stainless screws (SUS304). The grafting materials (hCP sheets and/or PRF) were put in the chamber as hCP sheets positioned at the bottom of the chamber. The skin flap was replaced and sutured. The care and use of animals followed the Guiding Principles for the Care and Use of Animals, as approved by Niigata University.

### 2.5 Microcomputed tomography analysis

To evaluate the volume of the mineral deposition and the newly formed bone, the implanted periosteal sheets and the harvested calvarial bone were scanned with a μCT scanner (SMX‐100CT; Shimadzu, Kyoto, Japan) as described previously (Kawase et al, 2011). The X‐ray beam conditions were 64 kV, 70 μA, field of view 16 m, and voxel size of 4 μm. The area of interest was reconstructed by using 3‐dimensional software (VGStudio Max 2; Volume Graphics GmbH, Heidelberg, Germany).

### 2.6 Histological processing and staining

For the samples implanted in nude mice, at 4 weeks after surgery, the mice were humanely killed by anesthetic overdose. The implanted periosteal sheets and PRF were harvested with surrounding tissues and fixed in 10% neutral‐buffered formalin for 24 hours. Those fixed samples, with extra tissues removed, were decalcified by using 0.5M ethylenediaminetetraacetic acid solution for 24 hours and then embedded in paraffin. Four‐μm‐thick sections were sliced and then stained with Masson's trichrome and hematoxylin and eosin. Some sections of calvaria were assayed for cellular expression of tartrate‐resistant acid phosphatase with an ACP staining kit (Muto Chemicals, Tokyo, Japan) (Kawase et al, 2009).

### 2.7 Immunohistological staining

To evaluate cell proliferation and angiogenesis within the implantation site, the sections were incubated overnight with mouse monoclonal anti‐PCNA (1:100; Santa Cruz Biotechnology, Santa Cruz, CA, USA) and mouse monoclonal anti‐αSMA (1:100; Abcam, Cambridge, MA, USA) diluted with Immuno Shot Mild (Cosmobio, Tokyo, Japan). Peroxidase‐conjugated goat antimouse antibodies (1:100) were used as secondary antibodies. Diaminobenzidine (KPL, Inc., Gaithersburg, MD, USA) served as the chromogen.

### 2.8 Statistical analysis

Significant differences between the groups were analyzed by using Student *t* test or one‐way analysis of variance. *P* values <0.05 were considered significant.

## 3 RESULTS

### 3.1 Microcomputed tomography analysis of osteogenic ability of the combined human‐cultured periosteal sheet with platelet‐rich fibrin in the mice's subcutaneous tissues

**Figure 1:**
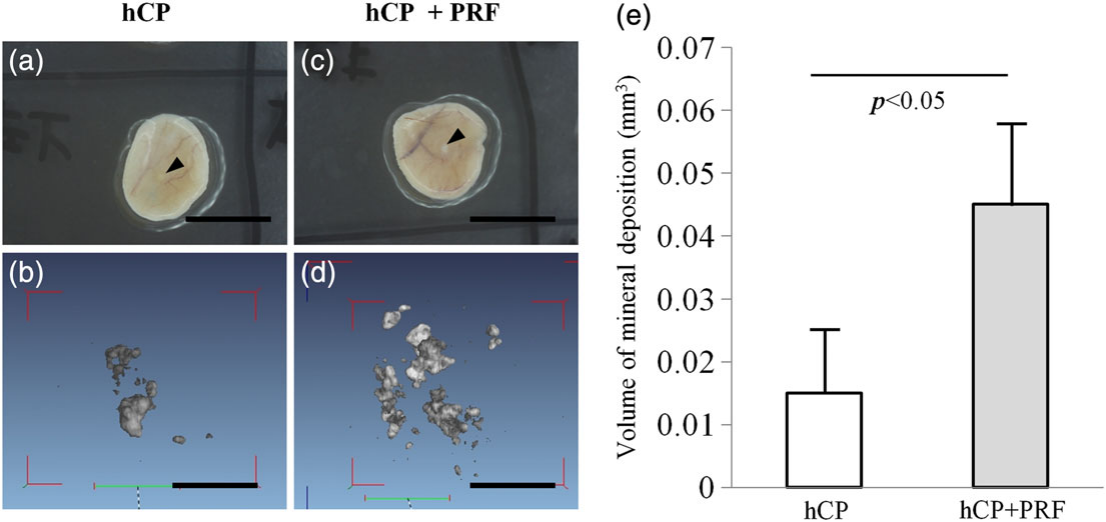
μCT analysis of specimens from subcutaneous tissues at 28 days postimplantation (hCP: n = 4, hCP + PRF: n = 4). Macroscopic images of the hCP sheet (a) and hCP + PRF (c). Arrow heads: the original periosteal segments. Bars: 8 mm. Three‐dimensional reconstructed μCT images of the hCP sheet (b) and hCP + PRF (d). Bars: 1 mm. (e) Volume of mineral deposition evaluated by μCT in implanted tissue

At 4 weeks postimplantation, mineral deposit formation of subcutaneously implanted grafting materials (Figure 1a and c) was evaluated by μCT. Radio‐opacity/mineral deposition was detected at the region within the implanted hCP sheets (Figure 1b). In the hCP combined with PRF, the radio‐opacity/mineral deposition was detected at the central region of the hCP sheets and surrounded marginal area of PRF (Figure 1d). The volume of mineral deposition (hCP vs hCP + PRF: 0.015 ± 0.010 mm^3^ vs 0.045 ± 0.013 mm^3^, *P <* 0.05; Figure 1e) increased significantly in the hCP + PRF complex implantation site compared with the hCP sheet alone.

### 3.2 Histological analyses of the mice's subcutaneous tissues

**Figure 2:**
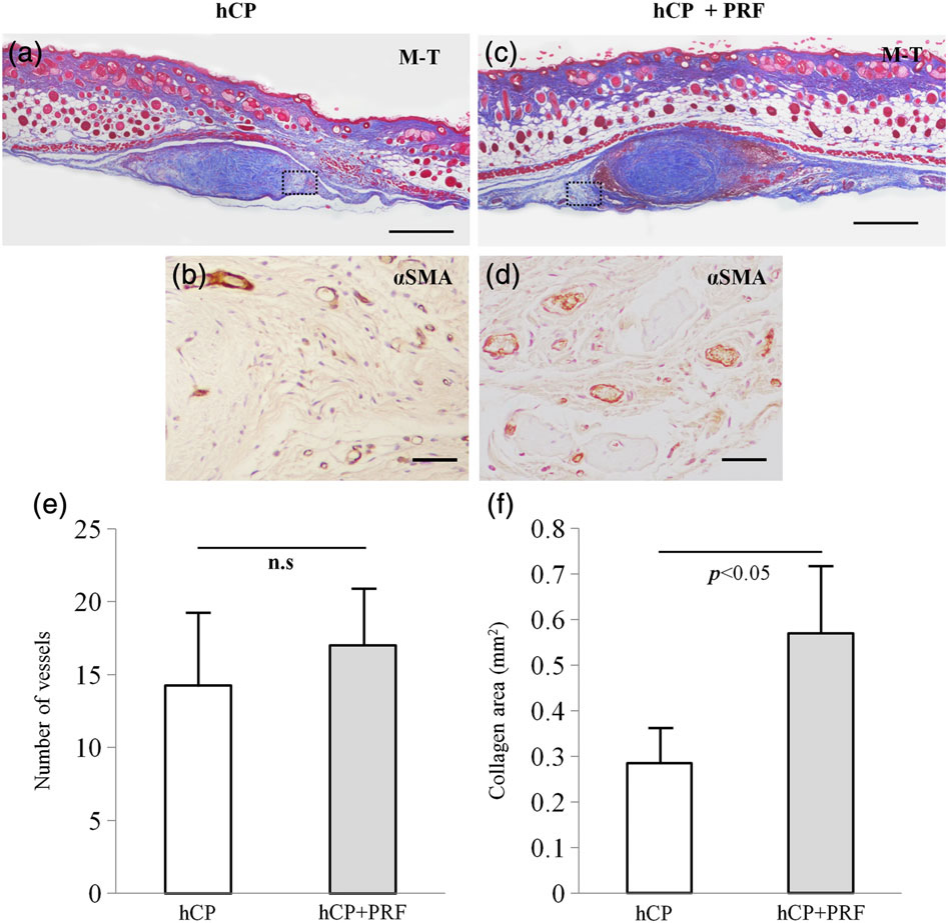
Histological analysis of specimens from subcutaneous tissues at 28 days postimplantation (hCP: n = 4, hCP + PRF: n = 4). Masson trichrome staining: hCP sheet (a) and hCP + PRF (c). Bars: 500 μm. Immunohistological staining for αSMA: hCP sheet (b) and hCP + PRF (d). Arrow heads: vessels characterized by αSMA‐positive endothelial cells. Bars: 50 μm. (e) Number of vessels per view. (f) Collagen area defined as blue collagen staining around the implanted periosteal segment

Platelet‐rich fibrin combined with hCP sheets stimulated the osteogenic potential of periosteal cells in vivo (figure S1). Furthermore, the implanted tissues were retrieved at 4 weeks postimplantation and examined histologically. The shape of the hCP sheets was maintained in subcutaneous tissues, and the collagen fibers were shown to extend from the periosteal segments (Figure 2a and c). The number of vessels in the region of the implantation site surrounded by the broken line (Figure 2b and d) was analyzed quantitatively, and there was no significant difference between the 2 groups (hCP vs hCP + PRF: 14.3 ± 4.9 vs 17.0 ± 3.92, *P* > 0.05; Figure 2e). There were significantly more collagen fibers around the segment (hCP vs hCP + PRF: 0.29 ± 0.08 mm^2^ vs 0.57 ± 0.15, *P* < 0.05; Figure 2f) in the hCP + PRF complex implantation site than in the hCP sheet alone.

### 3.3 Microcomputed tomography analysis of bone regeneration

**Figure 3:**
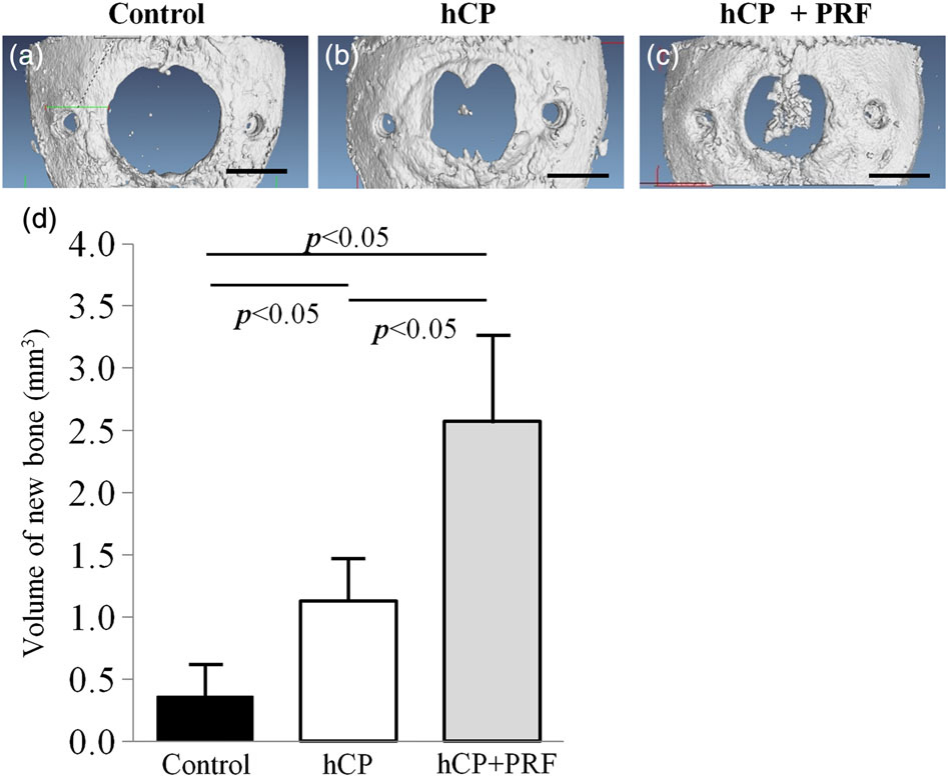
μCT analysis of bone regeneration in calvarial bone defects (control: n = 4, hCP: n = 4, hCP + PRF: n = 4). Three‐dimensional reconstructed μCT images of a calvarial bone defect at 28 days postimplantation: control (a), hCP sheet (b), and hCP + PRF (c). Bars: 2 mm. (d) Volume of new bone evaluated by μCT in a calvarial bone defect

The effect of the hCP + PRF complex on bone regeneration was further assessed in the mouse calvarial bone defects, which had bone metabolism. Figure 3a to c shows 3‐dimensional reconstructed images from each of 3 treatment groups (control, hCP, and hCP + PRF) at 4 weeks. In the sham control group, calvarial bone defects kept their original diameter (Figure 3a). New bone was observed at the central region within the periosteal segment in the hCP group (Figure 3b). The μCT images showed that the best regenerative response occurred in the hCP + PRF complex group (Figure 3c). Quantitative μCT analysis confirmed the volume of new bone formation in the bone defect at 4 weeks posttreatment (Figure 3d). There were significant increases of new bone formation in the hCP + PRF complex group (2.58 ± 0.68 mm^3^) compared with the hCP group (1.13 ± 0.34 mm^3^, *P* < 0.05) and the control group (0.36 ± 0.26 mm^3^, *P* < 0.05) and between the hCP group and the control group (*P* < 0.05).

### 3.4 Histological analyses of the calvarial defect

**Figure 4:**
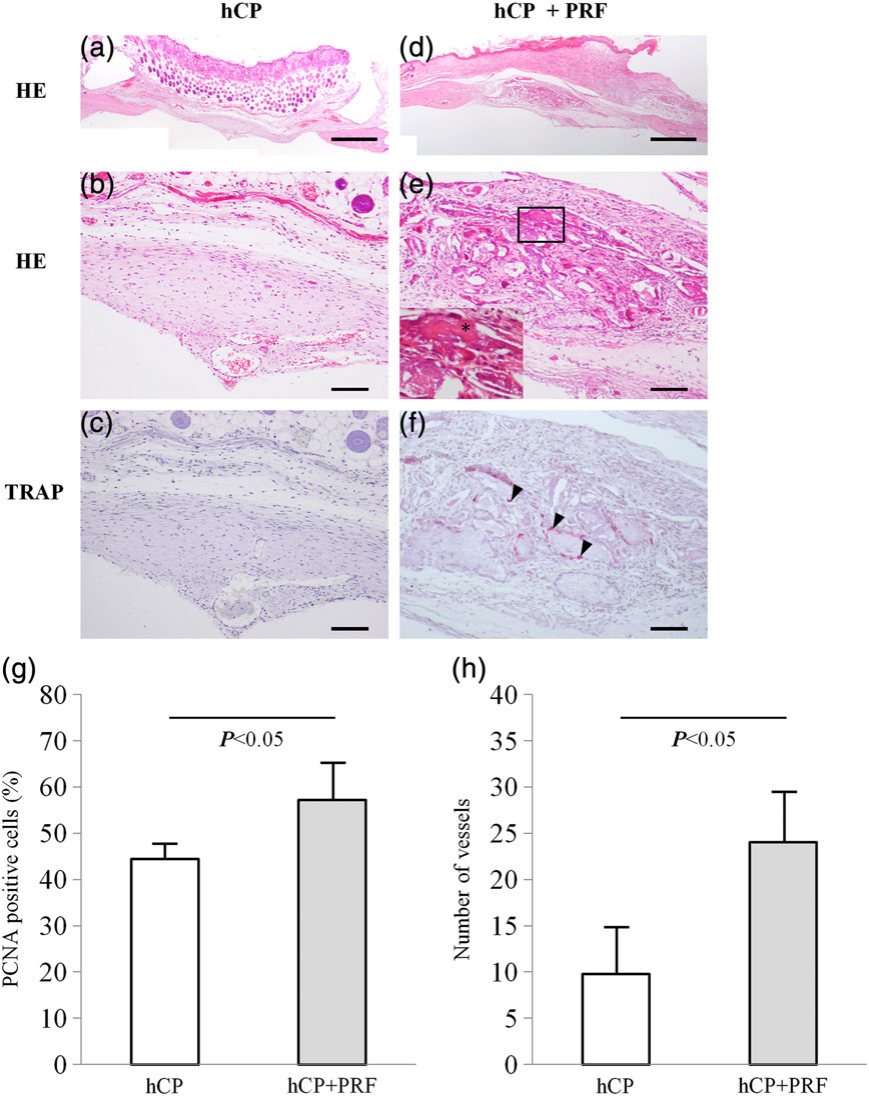
Histological analysis of specimens from implantation tissue in calvarial bone defects (hCP: n = 4, hCP + PRF: n = 4). H‐E staining and tartrate‐resistant acid phosphatase (TRAP) staining of sections taken from a defect site implanted with an hCP sheet (a‐c) or hCP + PRF complex (d‐f) at 28 days postimplantation. The asterisk (*) indicates a high magnification image of new bone. Arrow heads: TRAP‐positive osteoclast. Bars: 1 mm (a and d), 100 μm (b, c, e, and f). (g) Percentage of PCMA‐positive cells per view at the implanted site. (h) Number of vessels characterized by αSMA‐positive endothelial cells per view at the implanted site

New bone formation and tartrate‐resistant acid phosphatase‐positive osteoclasts were observed in the implantation material, especially in the site implanted with the hCP + PRF complex (Figure 4d, e, and f) compared with hCP alone (Figure 4a, b, and c). The rate of PCNA‐positive cells (hCP vs hCP + PRF: 44.4% ± 3.34% vs 57.2% ± 7.9%, *P* < 0.05) (Figure 4g) and the number of vessels (hCP vs hCP + PRF: 9.7 ± 4.9 vs 24.0 ± 5.6, *P* < 0.05) (Figure 4h) were significantly higher in the hCP + PRF complex implantation site than in the hCP alone group.

## 4 DISCUSSION

In the present study, the synergistic effects of PRF on bone regeneration were demonstrated in combined use with tissue engineered hCP sheets.

Platelet‐rich fibrin, an autologous platelet concentrate with fibrin clot, is considered to be a promising biomaterial in periodontal regeneration (Dohan Ehrenfest et al, 2009). Previous studies showed that the use of PRF might accelerate soft tissue regeneration and bone regeneration, while the effect of PRF with tissue engineered cells on cell‐based bone regenerative therapy was clinically limited (Roy et al, 2011; Yilmaz et al, 2014), indicating the deficient conditions of osteogenic cells in the grafting site. As in the present study, cell‐based medicine has been focused on bone tissue engineering, and the combination of PRF and osteogenic MSCs achieved excellent results (Wang et al, 2015), supporting our combination therapy with hCP.

The hCP sheet techniques make it possible to transplant a high density of cells rich in their endogenous extracellular matrix with intact cell‐cell and cell‐endogenous extracellular matrix contacts (Kawase et al, 2009). This characteristic is valuable for implantation without an exogenous scaffold, although the growth rate of the cell sheet is generally less than that of dispersed cells. In the present study, cell growth of periosteal cells was stimulated by combining the sheet with PRF in vivo. Platelet‐rich fibrin, as a source of growth factors such as PDGF and TGF‐β (Gassling et al, 2009), has been reported to promote proliferation of many types of cells (Dohan et al 2009), as well as periosteal cells in the present study. The release of growth factors was reported to last at least for 10 days (Kobayashi et al, 2016), and this property potentially promotes the proliferation of periosteal cells at implantation sites.

The preparation of PRF in this study by using commercially available centrifuge system was partially different to the standard PRF protocol developed by Choukroun and coworkers. The discrepancy between the methods should be concerned about biological property of PRF because the counts and distribution of platelet and major blood components contained in fibrin clots are dependent on centrifugal force (Watanabe et al, 2017). Recently, new modified protocols of PRF have been developed by Choukroun and coworkers (Fujioka‐Kobayashi et al, 2017; Ghanaati et al, 2014). Further experiments would be required to include various platelet formulations.

The fibrinogen and fibrin of PRF have been considered to be essential for initial cell adhesion and localization of periosteal cells (Gassling et al, 2013; Zhao et al, 2013). Platelet‐rich fibrin was found to stimulate human osteoblast differentiation and collagen production (Li et al, 2013). Because PRF is degraded by proteases for 1 to 2 weeks (Kawase et al, 2014), it may not be an ideal scaffold itself for bone regeneration. In the hCP‐PRF complex in the present study, the fibrin mesh of PRF was replaced by collagen with time in the subcutaneous implant. The hCP sheet and collagen could maintain the spaces required for tissue regeneration during the healing process.

Recently, the role of PRF to promote local angiogenesis was reported, indicating the effect of the contained VEGF (Li et al, 2013; Roy et al, 2011). Because angiogenesis is important for nutritional supply and recruitment of osteoblast precursors for bone regeneration (Song et al, 2013), stimulation of angiogenesis by PRF may activate the viability and proliferation of periosteal cells and facilitate bone regeneration.

Implantation of the hCP sheet and/or PRF into the mouse calvarial bone defects was performed and evaluated in bone tissue remodeling. The combined graft of hCP sheets and PRF showed approximately 2 to 3 times increased bone regeneration more than hCP alone, while the implantation of PRF alone was shown to promote limited bone regeneration of calvarial bone defects in the present study. The combined graft technique is expected to increase the bone regeneration in other different animal species and different defect sizes (Kang et al, 2011; Ozdemir et al, 2013; Pripatnanont et al, 2013).

It was suggested that PRF promoted the osteogenic ability of hCP sheets with implantation into animal subcutaneous tissues and calvarial defects, as well as increased new bone formation from host bone tissues. The growth factors and fibrin mesh of PRF could encourage proliferation and migration of host osteogenic cells into the bone defect (figure S2). Thus, the hCP + PRF complex seemed to have not only osteogenic capacity but also osteoinduction and osteoconduction capacity. Because these findings were limited in animal study, further human clinical trial needs to be performed using autologous hCP + PRF.

We have previously reported the effects of hCP sheets or PRF combined with synthetic bone on human bone regeneration in periodontal tissue, alveolar bone, and sinus grafting (Nagata et al, 2012, Okuda et al, 2015, Okuda et al, 2009, Yamamiya et al, 2008). The present study did not use any bone grafting materials to evaluate the real effects of the reciprocal communication between PRF and hCP sheet, in vitro and in vivo. To increase the predictability of the regenerative therapy, additional use of bone materials like autologous, xenogeneic, and/or synthetic bone graft seems to be important.

Therefore, further study is necessary to compare the synergistic effects of fresh combined use of hCP sheets (cells), PRF (growth factors), and bone graft materials (scaffolds).

## 5 CONCLUSION

In the limitation of the animal study, PRF could act as a biological scaffold for hCP sheets for bone regeneration therapy. The combined use of PRF and hCP sheets showed promise as an approach to grafting for periodontal bone regenerative therapy and alveolar ridge augmentation. Further elaborate investigations are necessary to provide the evidence to support clinical trials.

## CONFLICT OF INTEREST

The authors report no conflicts of interest related to this study.

## Supporting information

Figure S1. Histological observation of an hCP sheet and hCP‐PRF complex in vitro.

Figure S2. Active staining for alkaline phosphatase (ALP) of specimens from a calvarial bone defect site implanted with an hCP sheet or hCP sheet + PRF complex at 28 days postimplantation.

Figure S3. Supporting info item
