# Supplementary figures and images for: Synergistic effects of the combined use of human‐cultured periosteal sheets and platelet‐rich fibrin on bone regeneration: An animal study
Source: Clin Exp Dent Res. 2017 Aug 2;3(4):134–41. doi: 10.1002/cre2.71 (PMC5839211; doi:10.1002/cre2.71)

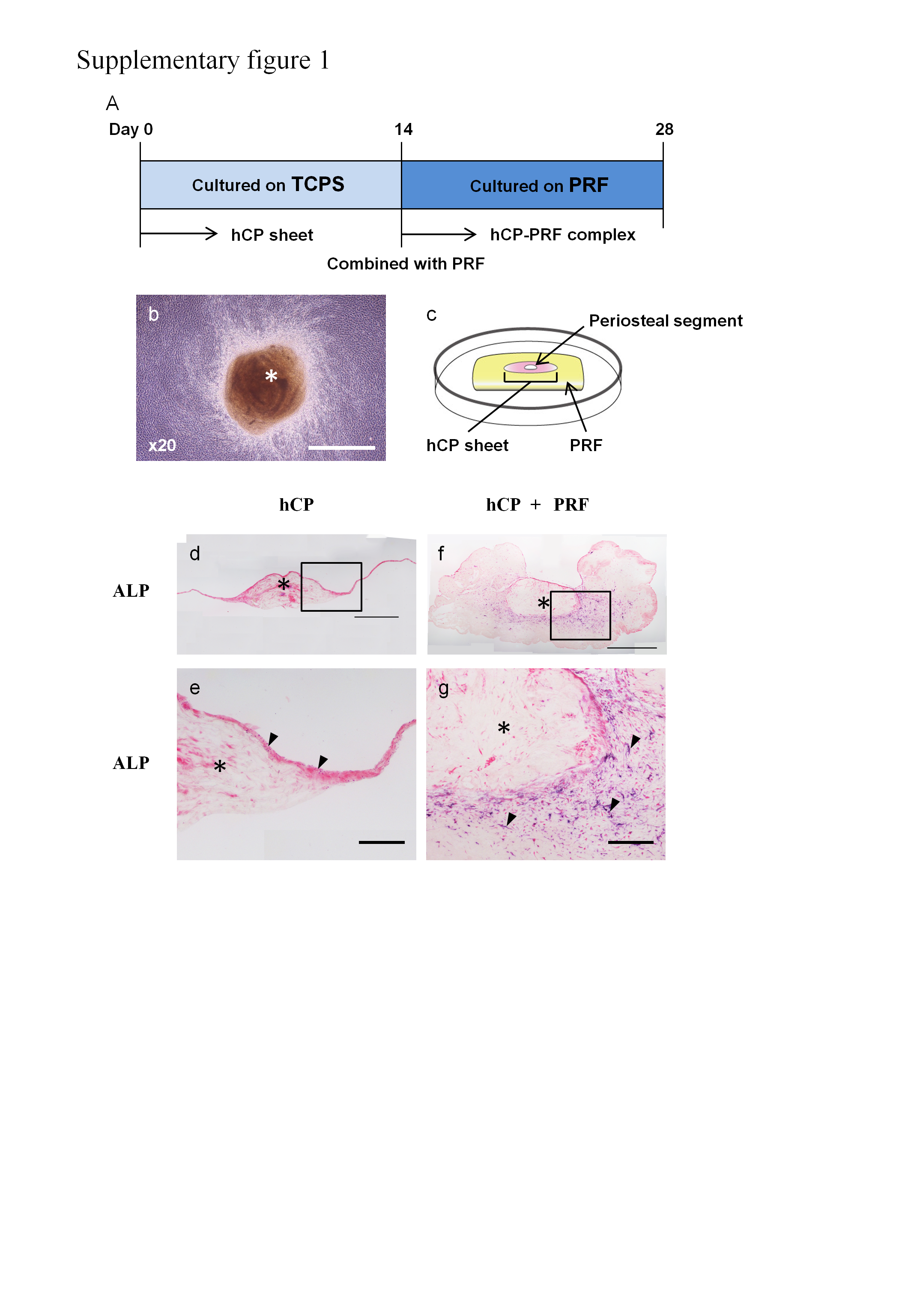

Supplement: Supplementary file 1 — Figure S1. Histological observation of an hCP sheet and hCP‐PRF complex in vitro. [file CRE2-3-134-s001.tif]

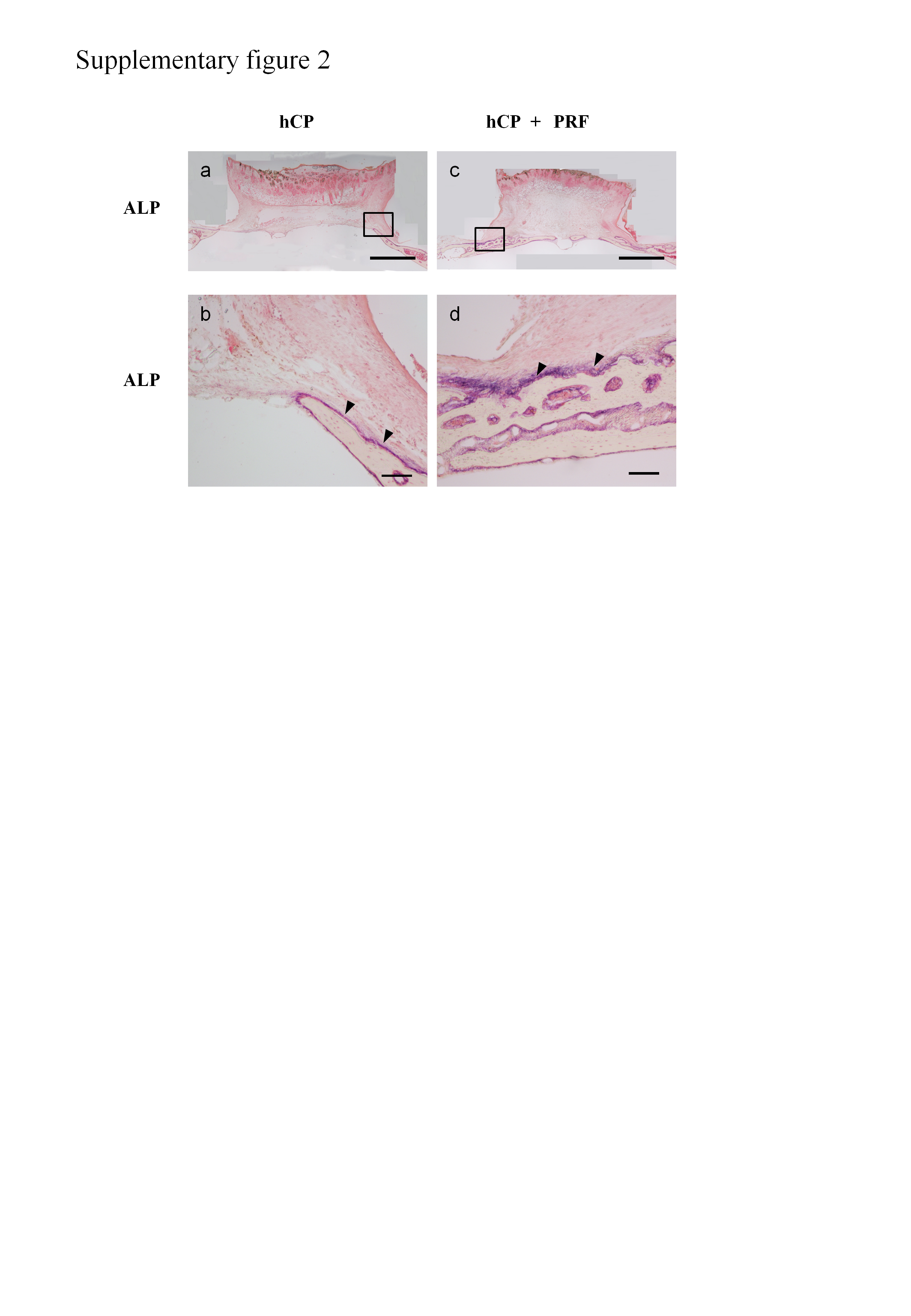

Supplement: Supplementary file 2 — Figure S2. Active staining for alkaline phosphatase (ALP) of specimens from a calvarial bone defect site implanted with an hCP sheet or hCP sheet + PRF complex at 28 days postimplantation. [file CRE2-3-134-s002.tif]
