# Supplementary material for: Synergistic effects of the combined use of human‐cultured periosteal sheets and platelet‐rich fibrin on bone regeneration: An animal study
Source: Clin Exp Dent Res. 2017 Aug 2;3(4):134–41. doi: 10.1002/cre2.71 (PMC5839211; doi:10.1002/cre2.71)
Supplement: Supplementary file 3 — Figure S3. Supporting info item [file CRE2-3-134-s003.doc]

**Supplementary Methods**

**Combining hCP sheets with PRF *in vitro***

The hCP sheets at 14 days were rinsed three times by PBS, harvested from the dish using a cell scraper while preserving cell sheet form, and placed on trimmed PRF (1 mm x 8 mm x 8 mm) (hCP-PRF complex) in the wet condition. Two mL of M199 medium were added to the dish, and the hCP-PRF complex was incubated for 12 h. The hCP-PRF complex was further cultured for 2 weeks. The medium was changed twice a week.

**Alkaline phosphatase (ALP) activity staining**

Some specimens were processed with cryohistological preparation for alkaline phosphatase (ALP) activity staining and immunohistological staining as described below. Cultured or hCP sheets with/without PRF implanted into the animals were fixed in 10% neutral-buffered formalin and decalcified using 0.5M EDTA solution for 24 hours. Thereafter, they were placed in 10-30% sucrose/PBS overnight and embedded in OTC compound (Sakura Finetek Japan, Tokyo, Japan) at -60°C. Frozen tissues were cryosectioned at seven-μm-thickness.

Cryosectioned samples were stained for ALP activity using an azo dye coupling technique with an ALP-staining kit (Sakura Finetek).

**Supplementary Figure legends**

**Supplementary figure 1**. Histological observation of an hCP sheet and hCP-PRF complex *in vitro*. **(A)** Time table for culture of hCP sheets and PRF preparations (TCPS: tissue culture polystyrene). **(B)** Light microscopy image of an hCP sheet cultured on a dish for 28 days. The asterisk (*) indicates the original periosteal tissue segment. Bars: 1 mm. **(C)** A scheme of the hCP-PRF complex. **(D-G)** Active staining for alkaline phosphatase (ALP) of an hCP sheet **(D, E)** at 28 days and the hCP+PRF complex **(F, G)** at 28 days (14 days after combination). Arrow heads: ALP-positive cells. (**D, E**: x40, **F, G**: x200). Asterisk: the original periosteal segment. Bars: 500 μm **(D, E)**; 100 μm **(F, G)**.

**Supplementary figure 2**. Active staining for alkaline phosphatase (ALP) of specimens from a calvarial bone defect site implanted with an hCP sheet **(A, B)** or hCP sheet+PRF complex **(A, C)** at 28 days post-implantation. Arrow heads: ALP-positive cells. Bars: 1 mm **(A, C)**, 50 μm **(B, D)**.
